# Prevalence and Social and Built Environmental Determinants of Maternal Prepregnancy Obesity in 68 Major Metropolitan Cities of the United States, 2013–2016

**DOI:** 10.1155/2021/6650956

**Published:** 2021-04-17

**Authors:** Gopal K. Singh, Jessica N. DiBari, Hyunjung Lee

**Affiliations:** ^1^US Department of Health and Human Service, Health Resources and Services Administration, Office of Health Equity, 5600 Fishers Lane, Rockville 20857, Maryland, USA; ^2^US Department of Health and Human Services, Health Resources and Services Administration, Maternal and Child Health Bureau, Office of Epidemiology & Research, Division of Research 5600 Fishers Lane, Rockville 20857, Maryland, USA; ^3^Health Resources and Services Administration, Office of Health Equity, Rockville, Maryland, USA; ^4^Oak Ridge Institute for Science and Education (ORISE), Oak Ridge, Tennessee, USA

## Abstract

**Objective:**

Maternal prepregnancy obesity is related to increased maternal morbidity and mortality and poor birth outcomes. However, prevalence and risk factors for prepregnancy obesity in US cities are not known. This study examines the prevalence and social and environmental determinants of maternal prepregnancy obesity (BMI ≥30), overweight/obesity (BMI ≥25), and severe obesity (BMI ≥40) in the 68 largest metropolitan cities of the United States.

**Methods:**

We fitted logistic and Poisson regression models to the 2013–2016 national vital statistics birth cohort data (*N* = 3,083,600) to derive unadjusted and adjusted city differentials in maternal obesity and to determine social and environmental determinants.

**Results:**

Considerable disparities existed across cities, with the prevalence of prepregnancy obesity ranging from 10.4% in San Francisco to 36.6% in Detroit. Approximately 63.0% of mothers in Detroit were overweight or obese before pregnancy, compared with 29.2% of mothers in San Francisco. Severe obesity ranged from 1.4% in San Francisco to 8.5% in Cleveland. Women in Anchorage, Buffalo, Cleveland, Fresno, Indianapolis, Louisville, Milwaukee, Oklahoma City, Sacramento, St Paul, Toledo, Tulsa, and Wichita had >2 times higher adjusted odds of prepregnancy obesity compared to those in San Francisco. Race/ethnicity, maternal age, parity, marital status, nativity/immigrant status, and maternal education were important individual-level risk factors and accounted for 63%, 39%, and 72% of the city disparities in prepregnancy obesity, overweight/obesity, and severe obesity, respectively. Area deprivation, violent crime rates, physical inactivity rates, public transport use, and access to parkland and green spaces remained significant predictors of prepregnancy obesity even after controlling for individual-level covariates.

**Conclusions:**

Substantial disparities in maternal prepregnancy obesity among the major US cities remain despite risk-factor adjustment, with women in several Southern and Midwestern cities experiencing high risks of obesity. Sound urban policies are needed to promote healthier lifestyles and favorable social and built environments for obesity reduction and improved maternal health.

## 1. Introduction

Obesity rates in the United States continue to rise unabated despite concerted efforts to reverse this trend [1–4]. About 42% of the US adult population is classified as obese (body mass index (BMI) ≥30 kg/m^2^) and 71% of the adult population is considered overweight or obese (BMI ≥25 kg/m^2^) [1, 2, 4]. The obesity prevalence of US women of childbearing age (18–49) increased nearly 4-fold, from 7.4% in 1976 to 27.5% in 2014; the overweight/obese prevalence rose from 22.8% in 1976 to 53.5% in 2014 [3, 5]. Maternal prepregnancy obesity and overweight rates have also risen in recent years, with 27.1% reporting obesity and 53.3% reporting overweight/obesity at the start of pregnancy in 2017 [5, 6].

There are multigenerational implications of this rising trend as they relate to maternal and child health [5]. Prepregnancy obesity is associated with an increased risk of chronic gestational diabetes, gestational hypertension, preeclampsia/eclampsia, cesarean section, and dysfunctional and prolonged labor [5–13]. Maternal prepregnancy obesity is considered an underlying cause of maternal mortality as it increases the risk of both direct and indirect obstetric causes of maternal death, including spontaneous abortion, hemorrhage, and uterine rupture [14–16]. Furthermore, maternal prepregnancy obesity can increase the risk of stillbirth, fetal macrosomia, preterm birth, selected birth defects, and infant mortality [5–12, 17]. Children of mothers who are overweight/obese are also at increased risk of obesity across the life course [8, 10, 12].

The socioecological perspective has been used as a framework to better understand the multiple determinants of obesity in terms of the social context and individual-level factors [18–20]. The social environment may encompass such community-level measures as poverty rate, income inequality, employment and labor market structure, housing quality, crime rate, public transport use, and access to grocery stores and healthy food [19–22]. The built environment refers to the physical structure, land uses, infrastructure planning, housing, sidewalks, walking paths, parks, and green spaces [19–25]. Although geographical disparities in adult and childhood obesity, particularly those by state and metropolitan areas, have been well documented, indicating the significance of various social and environmental factors as important determinants, such analyses of maternal prepregnancy obesity are limited [5, 20, 23, 26, 27]. Recent studies have shown state, regional, and rural-urban disparities in maternal prepregnancy obesity [5, 28]. However, the extent to which the prevalence of maternal prepregnancy obesity varies within urban or metropolitan areas is not known. More than 83% of the US population lives in urban or metropolitan areas, and as such, inequalities in adult obesity or prepregnancy obesity in urban areas largely drive the overall obesity trends and disparities in the US [27, 29].

Individual-level characteristics such as maternal age, parity, marital status, race/ethnicity, nativity/immigrant status, and education attainment have all been associated with prepregnancy obesity [5, 6, 9, 11]. City-level factors capturing the social and built environments at the community level may exert important influences on both individuals' risk of obesity and communities' obesity prevalence [20, 21]. For example, transportation systems may influence an individual's activity levels, where intricate transportation systems lend themselves to additional walking or physical activity [30]. Accessibility to fitness centers, grocery stores, and shopping centers may increase walking, resulting in lower obesity [31]. However, eating prepared foods at restaurants and fast food establishments may contribute to the upward weight trends. A study reported that exposure to fast food restaurants had a greater impact on lower socioeconomic status (SES) groups, resulting in higher obesity rates [32]. Safe neighborhoods with well-maintained sidewalks and walking and cycling paths are more supportive of an active lifestyle [30, 33]. Additional city-level factors, such as public school systems, poverty, income inequality, housing, crime rates, and access to parkland and green spaces, may also influence obesity and physical activity levels [20, 21, 23].

Despite the many adverse health effects of prepregnancy obesity on both the mother and child, geographic disparities in prepregnancy obesity and the many city-level factors contributing to obesity patterns have not been analyzed at the national level or among prepregnant and pregnant women. A better understanding of prepregnancy obesity risks and their determinants is critical to improving preconception health and health outcomes for both mothers and children [5].

The primary aim of this study was to examine city-specific patterns in prepregnancy obesity and overweight across 68 major metropolitan cities in the US and to identify key social and built environmental risk factors using national birth cohort data. The 68 selected cities account for 17% of the total US population and 21% of all US births [34, 35]. Health policy interventions to reduce obesity at the city level can decrease overall health disparities, improve intergenerational weight trends, and promote health equity across the United States, especially among populations and communities that are most vulnerable to suboptimal social and built environments [3, 5, 7, 20].

## 2. Methods

Maternal prepregnancy BMI data in this study were derived from the 2013, 2014, 2015, and 2016 national natality files [35, 36]. Only 69 of the largest cities, including New York, Los Angeles, Chicago, and Houston, were identified in the national files (Table 1). We excluded Honolulu from our analysis because of the small number of births. Information on prepregnancy height and weight has been collected on the birth certificate for selected states by the National Center for Health Statistics since 2003 [5, 35, 36]. The birth certificate data include the place of residence, such as state, county, and city of residence, a wide range of maternal and infant characteristics, medical risk factors and complications, and birth outcomes [35, 36]. Information on race/ethnicity, age, nativity/immigrant status, marital status, education, prepregnancy weight and height, and smoking before and during pregnancy is directly reported by the mother. Information on pregnancy complications and medical risk factors such as pregnancy-related hypertension and gestational diabetes is collected from the medical records at the hospital or the freestanding birthing center where the birth occurs [35, 36]. Detailed descriptions of the birth certificate data and national natality files are provided elsewhere [35, 36].

During 2013–2016, 3,083,600 births occurred among mothers living in the selected 68 cities. Of these mothers, 723,217 had prepregnancy obesity, 1,513,492 were overweight/obese, and 125,962 had severe obesity (BMI ≥40 kg/m^2^) at the start of pregnancy. Aggregating data for four years ensured sufficient sample sizes for analyzing obesity disparities across all 68 cities.

Besides the city of residence, we considered the following sociodemographic covariates of maternal prepregnancy obesity: maternal age, parity, race/ethnicity, nativity/immigrant status, marital status, and maternal education [5, 6, 9, 12, 28]. These covariates were measured as shown in Table 1. In addition to the individual-level covariates, we considered a number of city-level social and built environmental characteristics as having an impact on individuals' risk of obesity and on city-specific obesity rates. These included area deprivation, violent crime rate, physical inactivity rate, public transport use, access to parkland and green spaces, and air pollution [20, 21]. All city-level social and built environmental characteristics were linked to the individual birth records using the common geocode for cities.

We developed a factor-based deprivation index at the city level using the 2008–2012 American Community Survey (ACS) [34]. The deprivation index consisted of 11 socioeconomic indicators, which may be viewed as broadly representing educational opportunities, labor force skills, and economic and housing conditions in a given city [37, 38]. Selected indicators of education, occupation, wealth, income distribution, unemployment rate, poverty rate, and housing quality were used to construct the index by factor and principal components analyses [37, 38]. The factor loadings (correlations of indicators with the index) for the index varied from 0.96 for median family income to 0.66 for monthly housing costs. The deprivation index, measured as a continuous variable, had a mean of 100 and a standard deviation of 20, with higher index scores denoting higher levels of socioeconomic position and lower levels of deprivation [37, 38]. The index varied from a low socioeconomic score of 43.8 for Detroit and 53.4 for Cleveland to a high socioeconomic score of 144.0 for Seattle and 151.2 for San Francisco.

Access to parkland and green spaces was measured by the 2015 Park Score Index developed by the Trust for Public Land that combines data on the amount of parkland and green spaces, accessibility, investment, and park amenities [39]. Higher park scores indicate better access to and quality of parks, green space, and amenities. The Park Score Index ranged from a low score of 31.0 for Charlotte and 32.0 for Indianapolis to a high score of 84.0 for Minneapolis and St. Paul [39]. Data on violent crime rates were obtained from the 2015 Uniform Crime Report [40]. The violent crime rate was highest in St Louis (1817.1 per 100 000 population) and lowest in Virginia Beach (138.3). Data on public transport used for work commute were derived from the 2008–2012 ACS [34]. The percentage of population using public transport for commuting to work ranged from 0.2% in Arlington, Texas, to 55.6% in New York City. The 2015 physical inactivity rate, defined as the percentage of adults aged ≥18 years with no leisure-time physical activity, was derived from the CDC's 500 Cities Database [41]. Physical inactivity rates ranged from 14.3% in Seattle to 37.1% in Newark, New Jersey. Air pollution was measured by the 2015 data on the annual mean concentration of fine particulate matter, PM2.5 (µg/m^3^), which varied from a low of 4.7 micrograms per cubic meter for Tucson to 14.5 for Fresno [42].

Multivariable logistic regression models, estimated by the SAS LOGISTIC procedure, were used to derive differentials in individual risks of prepregnancy obesity, overweight/obesity, and severe obesity before and after adjusting for individual-level sociodemographic characteristics [43]. In estimating the odds of obesity for specific cities, we considered San Francisco as the reference because it had the lowest prevalence, which is potentially achievable by other cities. Secondly, using both individual- and city-level data, logistic models were fitted to assess the impact of city-level social and built environmental factors (measured as categorical variables: 1st quintile, 2nd–4th quintiles, and 5th quintile) on individual risks of prepregnancy obesity, overweight/obesity, and severe obesity before and after adjusting for individual-level covariates and city-fixed effects. Thirdly, city-level variations in the prevalence of obesity, overweight/obesity, and severe obesity were modeled as a function of city-level social and built environmental characteristics by Poisson regression models as estimated by the SAS GENMOD procedure [44]. Fitted logistic models were used to derive city-specific adjusted prevalence of obesity or overweight/obesity at mean values of the covariates. A relative index of disparity (RID) and coefficient of variation were used as summary measures of city disparities in obesity [20, 45].

No institutional review board (IRB) approval was required for this study, which is based on the secondary analysis of a public use federal database. However, the contents of the article, including methodological details, were reviewed and approved by the Health Resources and Services Administration's review committee.

## 3. Results

### 3.1. Prevalence and Individual-Level Risk Factors for Maternal Prepregnancy Obesity

During 2013–2016, the overall prevalence of maternal prepregnancy obesity in the 68 cities was 23.5%; the prevalence of overweight/obesity and severe obesity was 49.1% and 4.1%, respectively. Substantial disparities existed across cities, with the prevalence of prepregnancy obesity ranging from 10.4% in San Francisco and 13.4% in Seattle to 33.8% in Cleveland and 36.6% in Detroit (Table 1). Compared to San Francisco, all cities had 1.3 to 3.5 times higher prevalence of prepregnancy obesity (Table 1).

Cities varied greatly in their composition of sociodemographic characteristics known to be associated with obesity (Table 2). For example, the percentage of mothers aged ≥35 years was highest in San Francisco (38.3%) and Seattle (33.0%) and lowest in Toledo (8.9%). Educational attainment (percentage with a college degree) was highest among women in Seattle (65.6%) and San Francisco (65.4%) and lowest among women in Detroit (5.6%) and Cleveland (10.6%). The percentage of black mothers varied from 0.4% in Santa Ana and 2.4% in Anaheim to 71.5% in Memphis and 81.3% in Detroit. The percentage of Asian/Pacific Islander (API) mothers ranged from 1.2% in Miami and 1.4% in Detroit to 33.9% in San Francisco and 36.9% in San Jose. The percentage of nulliparous women (no previous birth) was highest in San Francisco (55.1%) and lowest in Fresno (32.1%). The percentage of women with four or more previous births was highest in Detroit (10.1%) and lowest in San Francisco (1.4%). The percentage of unmarried mothers ranged from 20.1% in San Francisco to 77.1% in Cleveland and 80.1% in Detroit. Approximately 5.2% of mothers in Toledo were foreign-born, compared with 60.9% in Miami.

A number of cities had markedly higher unadjusted odds of maternal prepregnancy obesity compared to San Francisco, most notably Detroit, Cleveland, and Milwaukee (Table 1, Model 1). Controlling for individual-level covariates reduced city differentials in obesity (Table 1, Model 2). Nevertheless, in the adjusted model, women in Anchorage, Buffalo, Cleveland, Fresno, Indianapolis, Louisville, Milwaukee, Oklahoma City, Sacramento, St. Paul, Toledo, Tulsa, and Wichita had more than two times higher odds of maternal prepregnancy obesity than those in San Francisco. Adjustment for individual-level covariates accounted for 63% of the city disparities in prepregnancy obesity (when comparing RID estimates in the unadjusted and adjusted models).

Table 1 shows the prevalence and odds of prepregnancy obesity according to other individual-level covariates. Compared to non-Hispanic whites, non-Hispanic black and AIAN women had, respectively, 119% and 80% higher adjusted odds of prepregnancy obesity, whereas API women had 30% lower adjusted odds. Increasing maternal age, higher parity, unmarried status, US-born status, and lower education were all associated with increased risks of maternal obesity. Women aged 40–44 and ≥ 45 years had 3.5 times higher adjusted odds of prepregnancy obesity than those aged <20 years. Women with four or more prior births had 43% higher adjusted odds of prepregnancy obesity than those who did not have prior births. Regarding joint effects of maternal age and parity, compared with nulliparity (no previous birth), parity ≥4 (four or more prior births) was associated with a 64% higher risk of prepregnancy obesity among women aged <20 years, a 94% higher risk of prepregnancy obesity among women aged 20–39 years, and a 107% higher risk of prepregnancy obesity among women aged ≥40 years (Table 3). US-born women had 85% higher adjusted odds of prepregnancy obesity than immigrant women. Women without a college degree had approximately twice the adjusted odds of prepregnancy obesity than those with a college degree (Table 1).

### 3.2. Prevalence and Individual-Level Risk Factors for Prepregnancy Overweight/Obesity and Severe Obesity

**Figure 1:**
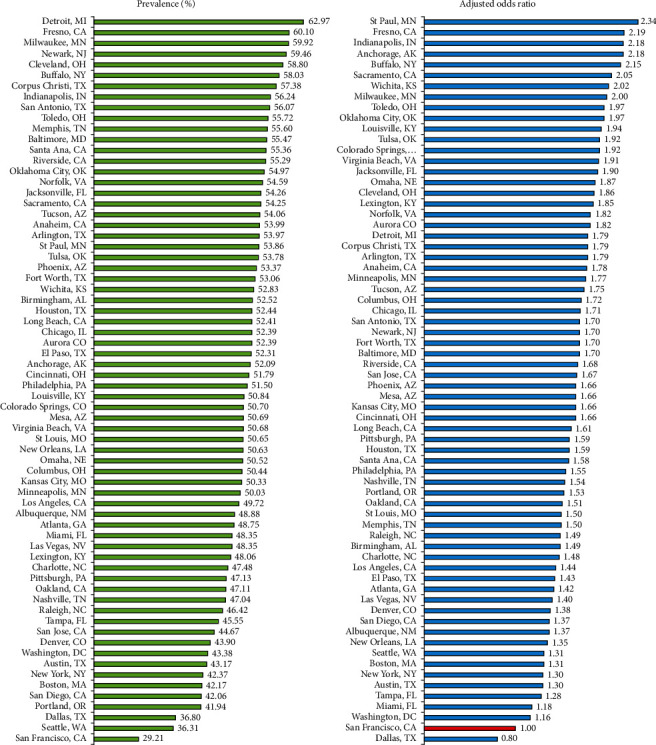
Prevalence^1^ and adjusted^2^ odds ratio for prepregnancy overweight/obesity (BMI ≥25) among women in 68 major US cities, 2013-2016 (*N* = 3,083,600). ^1^Prevalence estimates for all cities were significantly higher than the prevalence for San Francisco at *p* < 0.05. ^2^Adjusted by logistic regression for maternal age, race/ethnicity, parity, marital status, nativity/immigrant status, and maternal education (San Francisco as reference). All adjusted odds ratios were statistically significant at *p* < 0.05. Source: data derived from the 2013-2016 US National Natality data files.

City disparities in maternal prepregnancy overweight/obesity and severe obesity generally show patterns similar to those for prepregnancy obesity. The prevalence of prepregnancy overweight/obesity ranged from 29.2% in San Francisco and 36.3% in Seattle to 60.1% in Fresno and 63.0% in Detroit (Figure 1). Compared to women in San Francisco, women in Milwaukee, Wichita, Sacramento, Buffalo, Anchorage, Indianapolis, Fresno, and St. Paul had 2.0–2.3 times higher adjusted odds of overweight/obesity. Adjustment for individual-level covariates reduced city differentials in overweight/obesity by 39%.

**Figure 2:**
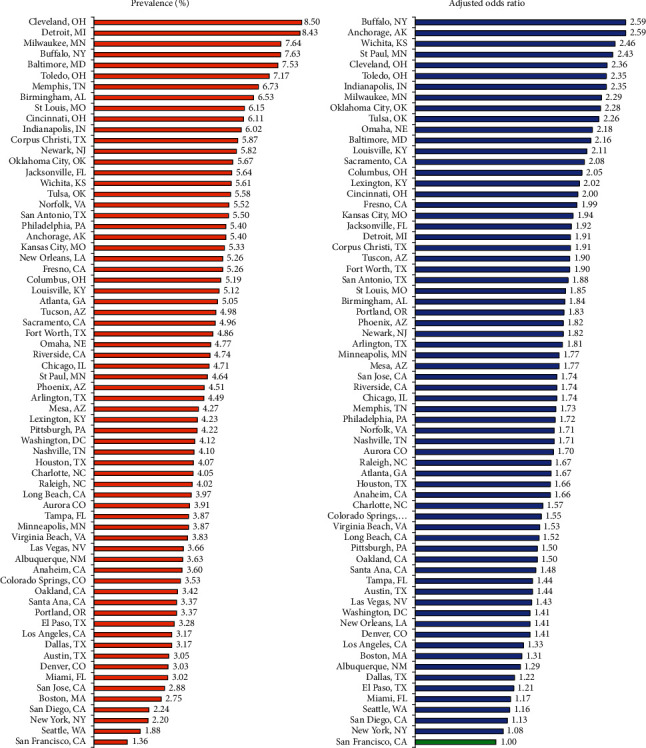
Prevalence^1^ and adjusted odds ratios^2^ for severe prepregnancy obesity (BMI ≥40) among women in 68 major US cities, 2013-2016 (*N* = 3,083,600). ^1^Prevalence estimates for all cities were significantly higher than the prevalence for San Francisco at *p* < 0.05. ^2^Adjusted by logistic regression for maternal age, race/ethnicity, parity, marital status, nativity/immigrant status, and maternal education (San Francisco as reference). All adjusted odds ratios except for New York were statistically significant at *p* < 0.05. Source: data derived from the 2013-2016 US National Natality data files.

The prevalence of severe obesity varied from 1.4% for women in San Francisco and 1.9% in Seattle to 8.4% for women in Detroit and 8.5% in Cleveland (Figure 2). Compared to San Francisco, the adjusted odds of severe obesity were 2.4 to 2.6 times higher in Indianapolis, Toledo, Cleveland, St. Paul, Wichita, Anchorage, and Buffalo. Adjustment for individual-level covariates accounted for 72% of the city differentials in severe obesity.

Estimating the Impact of City-Level Social and Built Environmental Characteristics on Prepregnancy Obesity, Overweight/Obesity, and Severe Obesity.

Women living in cities with high levels of socioeconomic deprivation (the lowest SES quintile) had 72% higher odds of prepregnancy obesity than those living in cities with low levels of socioeconomic deprivation (the highest SES quintile). After controlling for individual-level covariates, this differential was reduced to 16% higher odds among women in cities with high deprivation levels (Table 4). The association of area deprivation and overweight/obesity and severe obesity was similar to that for prepregnancy obesity.

Higher physical inactivity rates, lower public transport use, higher levels of air pollution, and lower access to parkland and green spaces were all independently associated with higher odds of prepregnancy obesity, overweight/obesity, and severe obesity even after controlling for individual-level covariates (Table 4). For example, compared to women in cities with high accessibility, women in cities with low accessibility to parkland and green spaces had 28%, 24%, and 34% higher adjusted odds of prepregnancy obesity, overweight/obesity, and severe obesity, respectively.

Table 5 shows the ecological associations between city-level social and built environmental factors and prepregnancy obesity prevalence. Cities with high levels of socioeconomic deprivation had 36%, 23%, and 48% higher risks of prepregnancy obesity, overweight/obesity, and severe obesity, respectively, after controlling for other city-level factors. Lower public transport use, higher levels of air pollution, and lower access to parkland and green spaces were all independently associated with a higher prevalence of prepregnancy obesity, overweight/obesity, and severe obesity at the city level. For example, controlling for other factors, cities with high violent crime rates, low transport use, and low access to parks and green spaces had, respectively, 71%, 45%, and 27% higher prevalence of severe prepregnancy obesity than cities with favorable social and built environments.

## 4. Discussion

To our knowledge, this is the first population-based study to examine maternal prepregnancy obesity among the largest US cities. The results of this study indicate substantial city disparities in the risk of prepregnancy obesity, overweight/obesity, and severe obesity, which were only partially explained by differences in maternal age, race/ethnicity, nativity, education, and other relevant sociodemographic characteristics. Estimating the city-specific maternal prepregnancy obesity prevalence and identifying individual- and city-level sociodemographic, behavioral, and environmental risk factors for obesity disparities across major cities are particularly novel features of our study.

We found the level of maternal prepregnancy obesity across the cities to be quite high, with the median prevalence being 25.4% for prepregnancy obesity and 51.7% for overweight/obesity. The magnitude of disparities was marked, with women in Detroit, Cleveland, Memphis, and Birmingham being at 3–6 times higher risks of prepregnancy obesity and severe obesity than their counterparts in San Francisco. Differences in maternal prepregnancy obesity in our highly urban sample according to individual-level covariates of maternal age, parity, race/ethnicity, nativity/immigrant status, and maternal education are generally consistent with those observed at the national level [5, 28]. Higher rates and risks of prepregnancy obesity and overweight associated with higher levels of area deprivation, physical inactivity rates, low use of public transport, lower access to parkland, green spaces, and neighborhood amenities, and higher levels of air pollution are compatible with those reported in prior research on adult and childhood obesity [20, 21, 26, 46].

The prevalence of prepregnancy obesity reported here for various cities is broadly consistent with the model-based estimates of obesity among adults aged ≥18 years, which indicate the highest rates of adult obesity in Detroit, Birmingham, Cleveland, Newark, Memphis, Milwaukee, and Buffalo and low rates of obesity in San Diego, Seattle, and San Francisco [41]. Our city-specific estimates of maternal prepregnancy obesity are also compatible with the city-related patterns in the adult prevalence of diabetes, hypertension, and coronary heart disease [41].

### 4.1. Implications for Health and Social Policy

This study supports the need to further explore the social and built environmental determinants of obesity. Health and social policy interventions can be tailored at the city level by identifying key aspects of the social and physical environment driving obesity trends that are amenable to change. To curb rising obesity rates, many federal programs have successfully advocated for policy and environmental changes to transform American communities into places that promote good nutrition and physical activity [21, 22]. Policymakers, public health officials, urban planners, social planners, and education administrators must work collaboratively to develop innovative public policy initiatives to influence the built environment, including the development of safe green spaces, walkable communities, accessible resources, and educational curriculum.

### 4.2. Limitations

Our study has some limitations. The individual-level analysis of obesity risks lacked data on important risk factors such as diet and physical activity of the mothers; other important SES measures such as family income, occupation, and employment status were not available in the national birth files. In the multilevel and ecological analyses of maternal prepregnancy obesity, the impact of the social and built environmental characteristics may have been underestimated as they are measured at the city level and do not account for the intracity heterogeneity in these characteristics. Neighborhoods or communities within a large city or an urban area can vary greatly in terms of access to parks and green spaces, sidewalks and walking paths, healthy foods and food security, safe and affordable housing, public transport, crime rates and public security, and exposure to air pollution and environmental hazards.

In our study, prevalence estimates of prepregnancy obesity and overweight included women who had a live birth during 2013–2016 and excluded women who became pregnant but experienced fetal loss, miscarriages, or abortions [5, 35, 36]. Since prepregnancy obesity in women is associated with these adverse perinatal outcomes, the reported prevalence of prepregnancy obesity is likely underestimated [5, 6]. Additionally, since prepregnancy weight on the birth certificate is self-reported by the mothers, prepregnancy obesity and overweight prevalence is likely to be underestimated [5, 6].

## 5. Conclusions

Our study shows substantial disparities in maternal prepregnancy obesity among major US cities, with women in several Southern and Midwestern cities experiencing a particularly high risk of obesity. Social and public health policies can help modify the social and built environments that are shown here to have a significant impact on maternal prepregnancy obesity [20–22]. Sound urban policies aimed at promoting healthier environments and lifestyles can address various aspects of the unfavorable social environment such as socioeconomic deprivation, poor housing, crime and public safety concerns, lack of access to parks, walking paths, green spaces, and grocery stores carrying healthy foods, lack of opportunities for recreation and physical activity, inadequate public transport, and exposure to environmental pollutants [20–22, 47]. Such policies not only are beneficial in terms of promoting increased physical activity, better nutrition, and reduced obesity levels among women, but can also provide other maternal health benefits such as improved physical and mental health and lower risks of chronic diseases, including heart disease, stroke, diabetes, and cancer [20–22, 47].

## Figures and Tables

**Table 1:** Observed prevalence and logistic regressions showing unadjusted and adjusted differentials in prepregnancy obesity (BMI ≥30) among women in 68 major cities and by selected socidemographic characteristics, United States, 2013–2016 (*N* = 3,083,600).

Covariate	Number of births	Prevalence percentage	Prevalence ratio	Model 1^1^			Model 2^2^			Covariate-adjusted	
				OR	95%	CI	OR	95%	CI	Prevalence	SE
City of residence											
Albuquerque, NM	28,367	22.79	2.19^*∗*^	2.54	2.43	2.66	1.35	1.29	1.41	20.90	0.23
Anaheim, CA	18,327	25.05	2.41^*∗*^	2.88	2.74	3.02	1.74	1.66	1.83	25.17	0.31
Anchorage, AK	18,137	26.62	2.56^*∗*^	3.12	2.97	3.28	2.30	2.19	2.42	30.28	0.35
Arlington, TX	22,310	26.68	2.56^*∗*^	3.13	2.99	3.28	1.83	1.75	1.92	26.08	0.28
Atlanta, GA	30,536	24.52	2.36^*∗*^	2.80	2.68	2.92	1.52	1.45	1.59	22.81	0.23
Aurora, CO	21,681	24.87	2.39^*∗*^	2.85	2.72	2.99	1.81	1.72	1.89	25.79	0.29
Austin, TX	56,870	18.98	1.82^*∗*^	2.02	1.94	2.10	1.35	1.29	1.41	20.96	0.17
Baltimore, MD	33,685	31.15	2.99^*∗*^	3.89	3.73	4.06	1.88	1.80	1.97	26.52	0.22
Birmingham, AL	13,101	28.94	2.78^*∗*^	3.50	3.33	3.69	1.68	1.59	1.77	24.49	0.34
Boston, MA	28,857	18.58	1.78^*∗*^	1.96	1.87	2.06	1.38	1.31	1.45	21.29	0.24
Buffalo, NY	14,092	32.44	3.12^*∗*^	4.13	3.93	4.34	2.29	2.17	2.41	30.17	0.37
Charlotte, NC	48,354	22.45	2.16^*∗*^	2.49	2.39	2.60	1.52	1.46	1.59	22.89	0.19
Chicago, IL	1,42,016	25.55	2.45^*∗*^	2.95	2.85	3.06	1.70	1.64	1.77	24.68	0.11
Cincinnati, OH	26,312	27.46	2.64^*∗*^	3.26	3.11	3.40	1.78	1.70	1.86	25.49	0.26
Cleveland, OH	22,632	33.81	3.25^*∗*^	4.39	4.20	4.60	2.01	1.91	2.10	27.69	0.28
Colorado Springs, CO	27,959	23.01	2.21^*∗*^	2.57	2.46	2.69	1.75	1.67	1.84	25.29	0.26
Columbus, OH	47,133	25.14	2.41^*∗*^	2.89	2.77	3.01	1.79	1.72	1.87	25.64	0.20
Corpus Christi, TX	17,972	29.93	2.88^*∗*^	3.67	3.50	3.85	1.78	1.69	1.87	25.54	0.30
Dallas, TX	86,638	16.97	1.63^*∗*^	1.76	1.69	1.83	0.96	0.92	1.00	16.09	0.12
Denver, CO	38,746	19.39	1.86^*∗*^	2.07	1.98	2.16	1.40	1.34	1.46	21.52	0.21
Detroit, MI	34,939	36.59	3.51^*∗*^	4.96	4.76	5.17	1.86	1.78	1.94	26.33	0.21
El Paso, TX	48,031	24.11	2.32^*∗*^	2.73	2.62	2.85	1.40	1.34	1.46	21.53	0.18
Fort Worth, TX	52,726	26.65	2.56^*∗*^	3.13	3.00	3.25	1.78	1.71	1.86	25.59	0.18
Fresno, CA	38,564	31.61	3.04^*∗*^	3.98	3.82	4.14	2.17	2.08	2.27	29.21	0.22
Houston, TX	2,03,061	24.50	2.35^*∗*^	2.79	2.69	2.90	1.59	1.53	1.65	23.59	0.09
Indianapolis, IN	13,598	29.50	2.83^*∗*^	3.60	3.42	3.79	2.22	2.10	2.34	29.59	0.38
Jacksonville, FL	42,901	28.20	2.71^*∗*^	3.38	3.24	3.52	1.92	1.84	2.01	26.92	0.21
Kansas City, MO	27,763	25.87	2.49^*∗*^	3.00	2.87	3.14	1.77	1.70	1.86	25.48	0.25
Las Vegas, NV	78,005	22.86	2.20^*∗*^	2.55	2.45	2.65	1.46	1.40	1.52	22.18	0.14
Lexington, KY	16,130	23.55	2.26^*∗*^	2.65	2.52	2.79	1.97	1.87	2.07	27.36	0.36
Long Beach, CA	22,757	24.95	2.40^*∗*^	2.86	2.73	3.00	1.60	1.53	1.68	23.73	0.27
Los Angeles, CA	1,91,719	22.94	2.20^*∗*^	2.56	2.47	2.66	1.48	1.42	1.54	22.41	0.09
Louisville, KY	9,298	26.09	2.51^*∗*^	3.04	2.87	3.22	2.04	1.93	2.17	28.06	0.46
Memphis, TN	38,585	30.46	2.93^*∗*^	3.77	3.62	3.93	1.61	1.54	1.68	23.63	0.20
Mesa, AZ	21,316	25.10	2.41^*∗*^	2.88	2.75	3.02	1.74	1.66	1.83	25.18	0.29
Miami, FL	41,988	20.51	1.97^*∗*^	2.22	2.13	2.32	1.17	1.12	1.22	18.79	0.18
Milwaukee, MN	38,050	33.78	3.24^*∗*^	4.39	4.21	4.57	2.11	2.02	2.20	28.63	0.22
Minneapolis, MN	23,127	22.89	2.20^*∗*^	2.55	2.44	2.68	1.72	1.64	1.80	24.93	0.28
Nashville, TN	9,457	22.77	2.19^*∗*^	2.54	2.39	2.69	1.65	1.55	1.75	24.20	0.43
New Orleans, LA	19,446	25.76	2.47^*∗*^	2.98	2.85	3.13	1.39	1.32	1.46	21.36	0.27
New York, NY	4,61,957	17.52	1.68^*∗*^	1.83	1.76	1.89	1.28	1.23	1.33	20.12	0.06
Newark, NJ	6,704	30.70	2.95^*∗*^	3.81	3.58	4.06	1.75	1.64	1.87	25.26	0.48
Norfolk, VA	13,782	27.54	2.65^*∗*^	3.27	3.11	3.44	1.71	1.63	1.81	24.88	0.35
Oakland, CA	19,840	21.33	2.05^*∗*^	2.33	2.22	2.45	1.52	1.45	1.60	22.90	0.29
Oklahoma City, OK	33,818	28.93	2.78^*∗*^	3.50	3.36	3.65	2.11	2.02	2.20	28.64	0.24
Omaha, NE	29,659	24.52	2.36^*∗*^	2.79	2.67	2.92	1.91	1.82	2.00	26.78	0.26
Philadelphia, PA	84,625	26.45	2.54^*∗*^	3.09	2.98	3.22	1.62	1.56	1.69	23.94	0.14
Phoenix, AZ	64,541	26.57	2.55^*∗*^	3.11	2.99	3.24	1.75	1.68	1.82	25.20	0.16
Pittsburgh, PA	7,464	23.08	2.22^*∗*^	2.58	2.42	2.75	1.60	1.50	1.71	23.69	0.49
Portland, OR	34,291	18.28	1.76^*∗*^	1.92	1.84	2.01	1.57	1.50	1.64	23.36	0.24
Raleigh, NC	23,905	22.17	2.13^*∗*^	2.45	2.34	2.57	1.59	1.52	1.67	23.61	0.27
Riverside, CA	22,451	27.93	2.68^*∗*^	3.33	3.19	3.49	1.74	1.66	1.82	25.15	0.27
Sacramento, CA	42,381	27.05	2.60^*∗*^	3.19	3.06	3.32	2.04	1.96	2.13	28.06	0.22
San Antonio, TX	94,710	29.81	2.86^*∗*^	3.65	3.52	3.79	1.83	1.76	1.91	26.06	0.13
San Diego, CA	74,081	17.40	1.67^*∗*^	1.81	1.74	1.89	1.32	1.27	1.38	20.63	0.15
San Francisco, CA	33,170	10.41	1.00	1.00	Reference		1.00	Reference		16.67	0.24
San Jose, CA	51,265	19.76	1.90^*∗*^	2.12	2.03	2.21	1.74	1.67	1.81	25.13	0.20
St Louis, MO	17,938	26.20	2.52^*∗*^	3.05	2.91	3.21	1.56	1.48	1.64	23.24	0.30
St Paul, MN	19,195	25.77	2.48^*∗*^	2.99	2.85	3.13	2.29	2.18	2.40	30.18	0.33
Tampa, FL	36,636	20.85	2.00^*∗*^	2.27	2.17	2.37	1.30	1.24	1.36	20.34	0.20
Toledo, OH	16,550	30.60	2.94^*∗*^	3.79	3.62	3.98	2.06	1.96	2.16	28.16	0.34
Tulsa, OK	24,925	27.59	2.65^*∗*^	3.28	3.13	3.43	2.01	1.91	2.10	27.69	0.28
Tucson, AZ	26,507	27.31	2.62^*∗*^	3.23	3.09	3.38	1.80	1.72	1.88	25.73	0.25
Virginia Beach, VA	18,548	23.20	2.23^*∗*^	2.60	2.47	2.73	1.73	1.64	1.82	25.00	0.32
Washington, DC	36,486	20.47	1.97^*∗*^	2.21	2.12	2.31	1.24	1.18	1.30	19.65	0.20
Wichita, KS	23,816	27.94	2.68^*∗*^	3.34	3.19	3.49	2.22	2.12	2.32	29.60	0.29

Race/ethnicity											
Non-Hispanic White	999736	16.63	1.00	1.00	Reference		1.00	Reference		16.65	0.04
Non-Hispanic Black	702024	33.43	2.01^*∗*^	2.52	2.50	2.54	2.19	2.17	2.21	29.68	0.06
American Indian/AN	15818	34.08	2.05^*∗*^	2.59	2.51	2.68	1.80	1.74	1.87	25.99	0.32
Asian/Pacific Islander	294256	8.72	0.52^*∗*^	0.48	0.47	0.49	0.70	0.69	0.71	12.32	0.07
Hispanic	1071766	27.18	1.63^*∗*^	1.87	1.86	1.88	1.98	1.96	2.00	27.74	0.05

Maternal age (years)											
<20	1,97,009	15.98	1.00	1.00	Reference		1.00	Reference		12.16	0.07
20–24	6,52,210	24.47	1.53^*∗*^	1.70	1.68	1.73	1.80	1.77	1.82	19.39	0.05
25–29	8,29,530	25.53	1.60^*∗*^	1.80	1.78	1.83	2.51	2.47	2.55	24.65	0.05
30–34	8,44,263	22.34	1.40^*∗*^	1.51	1.49	1.53	2.80	2.76	2.84	26.56	0.05
35–39	4,48,109	23.19	1.45^*∗*^	1.59	1.57	1.61	3.14	3.09	3.19	28.65	0.07
40–44	1,04,105	24.94	1.56^*∗*^	1.75	1.72	1.78	3.50	3.42	3.57	30.66	0.15
≥45	8,374	22.14	1.39^*∗*^	1.50	1.42	1.58	3.45	3.26	3.65	30.40	0.53

Parity											
0	12,49,953	18.07	1.00	1.00	Reference		1.00	Reference		21.02	0.04
1	9,29,988	23.18	1.289^*∗*^	1.36	1.35	1.37	1.17	1.17	1.18	23.66	0.04
2	4,92,818	28.65	1.599^*∗*^	1.81	1.80	1.82	1.28	1.27	1.30	25.20	0.06
3	2,23,547	33.1	1.839^*∗*^	2.23	2.21	2.25	1.39	1.38	1.41	26.63	0.09
≥4	1,75,646	36.08	2.009^*∗*^	2.55	2.52	2.57	1.43	1.41	1.45	27.11	0.10

Marital status											
Married	16,62,235	19.55	1.00	1.00	Reference		1.00	Reference		22.98	0.04
Unmarried	14,21,365	28.02	1.43^*∗*^	1.60	1.59	1.61	1.06	1.05	1.06	23.89	0.04

Nativity/immigrant status											
US-born	20,49,036	26.37	1.50	1.68	1.67	1.69	1.85	1.83	1.86	26.74	0.03
Foreign-born	10,25,745	17.62	1.00^*∗*^	1.00	Reference		1.00	Reference		17.06	0.04

Maternal education (years)											
<12	5,86,501	25.97	1.94^*∗*^	2.26	2.24	2.28	1.97	1.94	1.99	25.97	0.06
12	7,63,417	27.65	2.06^*∗*^	2.47	2.45	2.49	2.00	1.98	2.02	26.31	0.05
13–15	7,74,645	29.39	2.19^*∗*^	2.69	2.67	2.71	2.05	2.04	2.07	26.79	0.05
≥16	9,18,396	13.42	1.00	1.00	Reference		1.00	Reference		15.60	0.05

OR = odds ratio; CI = confidence interval; AN = Alaska Native. ^*∗*^Statistically significant at *p* < 0.05. ^1^Unadjusted for covariates. ^2^Adjusted for city of residence, race/ethnicity, maternal age, parity, marital status, nativity, and maternal education. Source: data derived from the 2013–2016 US National Natality data files.

**Table 2:** Differentials in sociodemographic risk factors for prepregnancy obesity and overweight analyses among US women in 68 major cities, United States, 2013–2016 (*N* = 3,083,600).

City of residence	Percentage of Asian/Pacific Islander population	Percentage of Black population	Percentage of Hispanic population	Percentage of population age <20 years	Percentage of population age ≥35 years	Percentage at parity 0 nulliparity	Percentage of women with parity ≥4	Percentage of unmarried mothers	Percentage of foreign-born mothers	Percentage of mothers with < high school	Percentage of college graduates
Albuquerque, NM	3.1	2.5	56.8	7.2	13.7	39.2	4.2	47.2	15.6	15.6	28.2
Anaheim, CA	14.6	2.4	65.1	6.6	18.7	37.2	5.0	41.4	49.3	22.8	21.8
Anchorage, AK	16.6	6.5	10.3	5.0	14.3	39.1	6.6	32.1	17.6	8.5	30.7
Arlington, TX	5.9	24.1	37.9	7.7	14.9	39.4	5.1	43.8	35.2	18.3	22.7
Atlanta, GA	6.0	51.9	13.3	6.0	20.0	41.2	6.8	51.1	22.7	17.0	43.2
Aurora CO	7.6	18.3	31.6	5.8	17.1	37.9	4.9	26.1	34.0	17.4	27.3
Austin, TX	8.5	7.7	43.9	5.9	21.3	42.8	4.5	35.4	33.3	19.8	42.3
Baltimore, MD	3.2	61.2	8.7	8.3	14.1	40.6	6.8	63.7	15.4	20.0	26.9
Birmingham, AL	2.4	60.0	8.0	7.3	11.7	38.5	5.1	57.5	11.8	15.5	27.8
Boston, MA	9.4	25.1	23.9	3.3	25.2	51.3	3.6	38.3	40.6	12.0	49.3
Buffalo, NY	6.6	39.7	13.8	9.9	11.8	39.1	7.9	65.3	17.1	23.8	20.0
Charlotte, NC	9.2	35.6	19.7	5.2	18.5	42.7	4.6	42.3	29.4	17.5	40.4
Chicago, IL	6.9	30.8	31.8	7.2	20.0	41.4	5.2	48.9	28.0	17.6	36.3
Cincinnati, OH	3.5	44.7	6.1	7.4	13.1	38.3	7.3	58.6	12.1	17.6	29.1
Cleveland, OH	2.3	58.4	11.6	11.4	9.1	36.5	8.4	77.1	8.9	26.4	10.6
Colorado Springs, CO	4.4	8.0	22.5	5.6	12.7	40.9	4.2	23.8	14.8	9.7	29.7
Columbus, OH	6.0	36.0	7.9	6.3	13.8	39.2	7.6	47.7	23.8	17.8	30.3
Corpus Christi, TX	2.2	3.2	68.7	10.5	11.5	36.7	5.5	53.9	11.0	18.7	17.5
Dallas, TX	4.2	23.2	50.5	9.3	15.2	44.2	5.1	51.3	37.7	24.3	22.8
Denver, CO	4.8	10.4	35.2	5.1	21.9	43.9	4.5	22.1	27.8	15.3	43.5
Detroit, MI	1.4	81.3	9.6	11.1	10.4	34.8	10.1	80.1	10.6	26.4	5.6
El Paso, TX	1.9	4.0	80.8	10.7	11.1	38.4	4.1	43.9	28.6	17.3	20.2
Fort Worth, TX	4.6	18.5	42.1	8.2	13.5	36.1	6.6	43.7	28.6	21.7	23.2
Fresno, CA	15.8	7.8	56.1	8.6	13.2	32.1	9.9	54.8	27.2	23.5	15.8
Houston, TX	7.7	21.9	52.2	8.1	15.8	38.0	5.7	47.3	44.5	29.3	25.1
Indianapolis, IN	4.5	31.0	14.2	8.3	11.9	39.8	6.8	54.0	18.9	22.1	25.8
Jacksonville, FL	5.5	37.2	10.2	5.9	13.4	39.2	5.3	47.8	17.6	12.5	25.3
Kansas City, MO	3.8	33.7	12.0	6.7	13.4	38.7	6.8	48.0	15.5	15.6	31.3
Las Vegas, NV	10.3	15.2	40.7	6.5	16.1	36.9	6.8	49.2	33.0	22.7	18.7
Lexington, KY	4.9	16.4	11.6	5.2	16.0	41.6	4.1	37.9	19.4	13.0	42.6
Long Beach, CA	14.4	13.3	52.7	5.9	19.6	41.0	4.8	49.1	32.4	19.7	25.7
Los Angeles, CA	9.4	8.5	61.1	6.1	23.1	40.2	5.0	48.8	46.2	25.3	26.9
Louisville, KY	3.6	27.3	5.7	8.0	12.5	43.9	4.4	47.8	12.7	12.7	32.5
Memphis, TN	1.9	71.5	10.2	11.1	10.0	34.7	9.1	71.9	12.0	22.7	16.1
Mesa, AZ	2.7	4.4	35.7	6.3	13.3	34.7	8.3	43.0	19.7	17.3	22.1
Miami, FL	1.2	32.6	55.5	5.5	19.3	42.5	4.5	58.3	60.9	14.2	19.7
Milwaukee, MN	6.5	48.4	20.6	9.3	11.9	34.2	9.8	67.0	17.7	22.7	17.4
Minneapolis, MN	7.8	30.4	12.0	4.5	21.3	41.9	8.7	39.9	31.0	20.3	43.7
Nashville, TN	4.7	30.0	14.7	5.9	14.7	40.8	4.4	41.5	26.6	18.8	35.4
New Orleans, LA	2.9	61.7	7.7	6.2	15.8	40.5	6.6	60.9	9.8	16.5	32.7
New York, NY	17.1	20.4	30.0	3.6	23.8	43.8	5.4	40.2	52.6	19.0	36.1
Newark, NJ	1.9	47.2	43.7	7.1	16.9	35.2	5.5	69.5	46.6	24.4	12.6
Norfolk, VA	3.8	46.6	9.2	6.4	10.1	42.5	5.8	46.4	10.7	10.5	22.3
Oakland, CA	19.2	21.5	31.2	4.3	27.9	46.0	3.7	40.6	38.3	19.0	38.4
Oklahoma City, OK	4.1	19.7	28.4	9.0	11.5	37.5	6.8	47.6	23.9	23.5	21.9
Omaha, NE	6.4	16.4	19.3	5.7	14.3	36.8	6.8	39.7	22.7	16.9	35.8
Philadelphia, PA	7.6	44.9	18.1	7.7	14.8	40.2	6.1	61.0	22.2	16.4	26.2
Phoenix, AZ	4.4	8.6	53.3	8.5	14.5	35.2	8.4	52.7	32.2	27.6	19.9
Pittsburgh, PA	4.2	34.0	2.3	5.2	15.5	44.5	4.5	52.5	8.7	8.4	40.3
Portland, OR	11.4	8.1	13.2	3.3	27.7	46.2	3.9	30.5	25.9	11.5	49.5
Raleigh, NC	5.3	30.4	18.2	4.5	20.6	43.0	4.1	36.3	26.1	14.2	46.8
Riverside, CA	5.5	5.2	65.4	6.7	14.8	36.4	5.9	44.7	29.6	20.6	19.0
Sacramento, CA	21.2	15.5	30.1	5.5	16.4	37.7	6.8	45.4	30.7	12.8	24.5
San Antonio, TX	3.4	6.8	68.0	9.1	13.0	36.6	6.4	46.7	20.2	20.0	21.1
San Diego, CA	18.6	6.7	36.0	3.8	22.9	45.2	3.2	28.0	37.3	9.2	45.1
San Francisco, CA	33.9	4.9	18.3	1.5	38.3	55.1	1.4	22.9	45.9	8.2	65.4
San Jose, CA	36.9	2.6	39.1	3.7	24.9	42.5	3.1	30.1	54.4	12.8	44.5
Santa Ana, CA	7.9	0.4	84.7	7.9	17.9	33.6	6.4	49.2	54.8	33.3	12.0
Seattle, WA	17.9	11.9	9.3	1.3	33.0	52.1	3.0	20.1	31.2	7.2	65.6
St Louis, MO	3.8	54.3	4.5	7.3	12.9	41.4	7.1	58.6	11.5	17.4	29.6
St Paul, MN	27.7	23.5	9.0	5.5	16.3	36.2	9.7	47.4	35.9	19.0	31.9
Tampa, FL	4.8	26.8	30.0	5.8	15.0	40.4	5.2	51.8	29.0	12.9	27.9
Toledo, OH	1.6	33.1	10.6	8.9	8.9	36.4	6.3	63.6	5.2	19.1	16.7
Tulsa, OK	4.8	19.5	21.3	8.7	11.5	35.7	6.6	50.7	22.5	26.0	20.3
Tucson, AZ	3.7	5.4	53.3	7.5	13.2	37.7	5.6	50.3	23.1	19.6	21.2
Virginia Beach, VA	6.8	22.1	10.0	3.2	15.4	42.5	3.1	29.4	14.3	3.8	35.8
Washington, DC	4.7	50.6	12.8	5.6	25.5	46.8	4.4	49.0	25.5	14.3	46.6
Wichita, KS	4.8	13.5	21.2	8.3	11.1	35.5	7.0	46.5	17.1	16.8	25.9

Source: Data derived from the 2013–2016 US National Natality data files.

**Table 3:** Joint effect of maternal age and parity on prepregnancy obesity (BMI ≥30), overweight/obesity (BMI ≥25), and severe obesity (BMI ≥40) among women in 68 major cities, United States, 2013–2016 (*N* = 3,083,600).

	Number of births	Prevalence percent	Prevalence ratio	
Prepregnancy obesity				
Maternal age <20 years				
Parity 0 (nulliparity)	1,60,476	15.10	1.00	Reference
Parity 1–3	35,635	19.96	1.32	^*∗*^
Parity 4+	93	24.73	1.64	^*∗*^

Maternal age 20–39 years				
Parity 0 (nulliparity)	10,59,867	18.52	1.00	Reference
Parity 1–3	15,45,945	26.38	1.42	^*∗*^
Parity 4+	1,57,851	35.98	1.94	^*∗*^

Maternal age ≥40 years				
Parity 0 (nulliparity)	29,610	17.94	1.00	Reference
Parity 1–3	64,773	24.45	1.36	^*∗*^
Parity 4+	17,702	37.08	2.07	^*∗*^

Prepregnancy overweight/obesity				
Maternal age <20 years				
Parity 0 (nulliparity)	1,60,476	37.61	1.00	Reference
Parity 1–3	35,635	45.91	1.22	^*∗*^
Parity 4+	93	51.61	1.37	^*∗*^

Maternal age 20–39 years				
Parity 0 (nulliparity)	10,59,867	41.58	1.00	Reference
Parity 1–3	15,45,945	53.53	1.29	^*∗*^
Parity 4+	1,57,851	65.34	1.57	^*∗*^

Maternal age ≥40 years				
Parity 0 (nulliparity)	29,610	41.45	1.00	Reference
Parity 1–3	64,773	53.25	1.28	^*∗*^
Parity 4+	17,702	70.04	1.69	^*∗*^

Prepregnancy severe obesity				
Maternal age <20 years				
Parity 0 (nulliparity)	1,60,476	1.83	1.00	Reference
Parity 1–3	35,635	2.28	1.25	^*∗*^
Parity 4+	93	5.38	2.94	

Maternal age 20–39 years				
Parity 0 (nulliparity)	10,59,867	3.25	1.00	Reference
Parity 1–3	15,45,945	4.67	1.44	^*∗*^
Parity 4+	1,57,851	6.85	2.11	^*∗*^

Maternal age ≥40 years				
Parity 0 (nulliparity)	29,610	2.90	1.00	Reference
Parity 1–3	64,773	3.65	1.26	^*∗*^
Parity 4+	17,702	5.29	1.82	^*∗*^

^*∗*^Statistically significant at *p* < 0.05. Source: data derived from the 2013–2016 US National Natality data files.

**Table 4:** Logistic models showing unadjusted and covariate-adjusted odds ratios (OR) for prepregnancy obesity, overweight/obesity, and severe obesity according to city-level social and environmental characteristics, United States, 2013–2016 (*N* = 3,083,600).

	Obesity (BMI ≥ 30)				Overweight/obesity (BMI ≥ 25)				Severe obesity (BMI ≥ 40)			
	Unadjusted		Covariate-adjusted^1^		Unadjusted		Covariate-adjusted^1^		Unadjusted		Covariate-adjusted^1^	
	OR	95% CI	OR	95% CI	OR	95% CI	OR	95% CI	OR	95% CI	OR	95% CI
Socioeconomic deprivation index, 2008–2012^2^												
43.85–86.09 (low SES)	1.72	1.71–1.74	1.16	1.15–1.17	1.60	1.58–1.61	1.13	1.12–1.14	2.01	1.97–2.05	1.22	1.19–1.25
86.10–112.64 (middle SES)	1.30	1.29–1.31	1.07	1.06–1.08	1.27	1.26–1.27	1.05	1.04–1.06	1.32	1.29–1.34	1.08	1.06–1.10
112.65–151.24 (high SES)	1.00	Reference	1.00	Reference	1.00	Reference	1.00	Reference	1.00	Reference	1.00	Reference

Violent crime rate/100,000 population, 2015												
138.30–478.29 (low crime)	1.00	Reference	1.00	Reference	1.00	Reference	1.00	Reference	1.00	Reference	1.00	Reference
478.30–1108.03	1.09	1.08–1.10	1.00	0.99–1.00	1.06	1.06–1.07	0.97	0.96–0.97	1.17	1.15–1.19	1.04	1.02–1.06
1108.04–1817.10 (high crime)	1.51	1.49–1.52	1.13	1.11–1.14	1.32	1.31–1.33	1.05	1.04–1.06	2.02	1.97–2.06	1.28	1.25–1.31

Physical inactivity (%), 2015^3^												
14.30–21.75 (low inactivity)	1.00	Reference	1.00	Reference	1.00	Reference	1.00	Reference	1.00	Reference	1.00	Reference
21.76–31.89	1.22	1.21–1.23	1.05	1.04–1.06	1.19	1.18–1.20	1.02	1.02–1.03	1.25	1.23–1.27	1.05	1.03–1.06
31.90–37.10 (high inactivity)	1.41	1.40–1.43	1.05	1.04–1.06	1.35	1.34–1.36	0.99	0.99–1.00	1.54	1.51–1.57	1.13	1.10–1.15

Public transport use for work commute (%), 2008–2012												
0.24–1.99 (low use)	1.39	1.37–1.40	1.29	1.28–1.30	1.33	1.32–1.34	1.28	1.27–1.29	1.49	1.46–1.52	1.14	1.11–1.16
2.00–12.03	1.26	1.25–1.27	1.11	1.10–1.12	1.22	1.22–1.23	1.08	1.08–1.09	1.31	1.30–1.33	1.11	1.09–1.14
12.04–55.59 (high use)	1.00	Reference	1.00	Reference	1.00	Reference	1.00	Reference	1.00	Reference	1.00	Reference

Park index score, 2015^4^												
31.00–42.49 (low access)	1.55	1.53–1.56	1.28	1.27–1.29	1.44	1.43–1.45	1.24	1.23–1.25	1.73	1.70–1.77	1.34	1.32–1.37
42.50–66.49	1.33	1.32–1.34	1.11	1.11–1.12	1.26	1.26–1.27	1.08	1.07–1.08	1.48	1.46–1.50	1.21	1.20–1.23
66.50–84.00 (high access)	1.00	Reference	1.00	Reference	1.00	Reference	1.00	Reference	1.00	Reference	1.00	Reference

Air pollution-annual mean PM2.5 (*μ*g/m^3^), 2015												
4.70–7.19 (low pollution)	1.00	Reference	1.00	Reference	1.00	Reference	1.00	Reference	1.00	Reference	1.00	Reference
7.20–9.69	0.94	0.93–0.95	1.01	1.00–1.02	0.91	0.90–0.92	0.99	0.98–0.99	0.98	0.96–0.99	1.03	1.02–1.05
9.70–14.50 (high pollution)	1.15	1.14–1.16	1.09	1.08–1.10	1.12	1.12–1.13	1.07	1.06–1.08	1.20	1.17–1.22	1.11	1.09–1.14

^1^Adjusted for individual-level covariates of race/ethnicity, maternal age, parity, marital status, nativity status, and maternal education. For all city-level covariates, the first category represents the first quintile, the second category represents the second through fourth quintiles, and the third category represents the fifth quintile. ^2^The socioeconomic deprivation index is a continuous variable with a mean of 100 and a standard deviation of 20. Higher socioeconomic index scores denote higher levels of socioeconomic position and lower levels of deprivation. ^3^No leisure-time physical activity among adults aged > = 18 years. ^4^The Index, developed by the Trust for Public Land, combines data on the amount of parkland and green spaces, accessibility, investment, and park amenities. Higher park scores indicate better access to and quality of parks, green space, and amenities.

**Table 5:** Poisson regression models showing relative risk (RR) of prepregnancy obesity, overweight/obesity, and severe obesity according to city-level social and environmental characteristics, United States, 2013–2016 (*N* = 68 cities).

	Obesity (BMI ≥30)				Overweight/obesity (BMI ≥25)				Severe obesity (BMI ≥40)			
	Unadjusted		Covariate-adjusted		Unadjusted		Covariate-adjusted		Unadjusted		Covariate-adjusted	
	RR	95% CI	RR	95% CI	RR	95% CI	RR	95% CI	RR	95% CI	RR	95% CI
Socioeconomic deprivation index, 2008–2012^1^												
43.85–86.09 (low SES)	1.53	1.52–1.54	1.36	1.35–1.36	1.28	1.28–1.29	1.23	1.22–1.24	2.01	1.97–2.05	1.48	1.44–1.52
86.10–112.64 (middle SES)	1.23	1.22–1.24	1.22	1.20–1.23	1.14	1.14–1.15	1.16	1.16–1.17	1.32	1.29–1.34	1.18	1.15–1.21
112.65–151.24 (high SES)	1.00	Reference	1.00	Reference	1.00	Reference	1.00	Reference	1.00	Reference	1.00	Reference

Violent crime rate/100,000 population, 2015												
138.30–478.29 (low crime)	1.00	Reference	1.00	Reference	1.00	Reference	1.00	Reference	1.00	Reference	1.00	Reference
478.30–1108.03	1.07	1.07–1.08	0.98	0.97–0.99	1.03	1.03–1.04	0.95	0.94–0.96	1.17	1.15–1.19	1.12	1.10–1.15
1108.04–1817.10 (high crime)	1.37	1.36–1.38	1.18	1.16–1.19	1.15	1.15–1.16	1.04	1.03–1.05	2.02	1.97–2.06	1.71	1.67–1.76

Physical inactivity (%), 2015^2^												
14.30–21.75 (low inactivity)	1.00	Reference	1.00	Reference	1.00	Reference	1.00	Reference	1.00	Reference	1.00	Reference
21.76–31.89	1.17	1.16–1.18	1.01	1.00–1.02	1.10	1.10–1.11	1.00	1.00–1.01	1.24	1.22–1.26	1.02	0.99–1.05
31.90–37.10 (high inactivity)	1.32	1.31–1.33	1.02	1.01–1.03	1.18	1.17–1.18	1.01	1.00–1.01	1.55	1.52–1.58	1.03	1.00–1.05

Public transport use for work commute (%), 2008–2012												
0.24–1.99 (low use)	1.29	1.28–1.30	1.24	1.23–1.26	1.17	1.16–1.17	1.14	1.14–1.15	1.49	1.46–1.52	1.45	1.41–1.48
2.00–12.03	1.20	1.20–1.21	1.08	1.08–1.09	1.12	1.11–1.12	1.05	1.05–1.06	1.31	1.30–1.33	1.11	1.09–1.14
12.04–55.59 (high use)	1.00	Reference	1.00	Reference	1.00	Reference	1.00	Reference	1.00	Reference	1.00	Reference

Park index score, 2015^3^												
31.00–42.49 (low access)	1.42	1.40–1.43	1.15	1.14–1.16	1.22	1.21–1.23	1.07	1.06–1.07	1.75	1.73–1.79	1.27	1.24–1.31
42.50–66.49	1.27	1.26–1.27	1.09	1.08–1.10	1.14	1.14–1.14	1.03	1.02–1.03	1.50	1.48–1.52	1.22	1.19–1.24
66.50–84.00 (high access)	1.00	Reference	1.00	Reference	1.00	Reference	1.00	Reference	1.00	Reference	1.00	Reference

Air pollution-annual mean PM2.5 (*μ*g/m^3^), 2015												
4.70–7.19 (low pollution)	1.00	Reference	1.00	Reference	1.00	Reference	1.00	Reference	1.00	Reference	1.00	Reference
7.20–9.69	0.95	0.95–0.96	1.01	1.01–1.02	0.95	0.95–0.96	0.99	0.98–0.99	0.98	0.96–0.99	1.06	1.04–1.08
9.70–14.50 (high pollution)	1.11	1.10–1.12	1.08	1.07–1.08	1.06	1.06–1.07	1.05	1.05–1.06	1.20	1.17–1.22	1.09	1.07–1.11

For all covariates, the first category represents the first quintile, the second category represents the second through fourth quintiles, and the third category represents the fifth quintile. ^1^The socioeconomic deprivation index is a continuous variable with a mean of 100 and a standard deviation of 20. Higher socioeconomic index scores denote higher levels of socioeconomic position and lower levels of deprivation. ^2^No leisure-time physical activity among adults aged > = 18 years. ^3^The Index, developed by the Trust for Public Land, combines data on the amount of parkland and green spaces, accessibility, investment, and park amenities. Higher park scores indicate better access to and quality of parks, green space, and amenities.

## Data Availability

Datasets used to analyze and support the findings of this study are cited in the article and can be obtained from US Centers for Disease Control and National Center for Health Statistics.
